# New Insights into the Production of Assyrtiko Wines from the Volcanic Terroir of Santorini Island Using *Lachancea thermotolerans*

**DOI:** 10.3390/microorganisms12040786

**Published:** 2024-04-12

**Authors:** Aikaterini Tzamourani, Spiros Paramithiotis, Marion Favier, Joana Coulon, Virginie Moine, Ioannis Paraskevopoulos, Maria Dimopoulou

**Affiliations:** 1 Department of Wine, Vine and Beverage Sciences, School of Food Science, University of West Attica, 28 Ag. Spyridonos St., 12243 Egaleo, Greece; 2 Department of Biological Applications and Technology, University of Ioannina, 45110 Ioannina, Greece; 3 BioLaffort, 11 rue Aristide Bergès, 33270 Floirac, Francejoana.coulon@laffort.com (J.C.); virginie.moine@laffort.com (V.M.); 4 GAIA Wines, 84700 Santorini, Greece

**Keywords:** climate change, Assyrtiko wines, microbial interactions, *Lachancea thermotolerans*, perception interactions, acid composition

## Abstract

Assyrtiko is a rare ancient grape variety of Greece, which is known to produce Protected Designation of Origin (PDO) Santorini white wines. Besides the famous character of the volcanic terroir, Assyrtiko of Santorini is also marked by a low pH value and sharp acidity. The aim of the present study was to apply a new inoculation procedure that modulates the fermentation process by maintaining the unique sensorial characteristics of Assyrtiko wines based on acidity. For this purpose, the *Lachancea thermotolerans* species, known for the formation of lactic acid, was tested in sequential fermentation with three different *Saccharomyces cerevisiae* strains. At the end of the fermentation process, implantation control for *S. cerevisiae* strains (interdelta sequence profile analysis) was performed, oenological parameters were determined according to the OIV protocols, and the volatile compounds produced were measured by gas chromatography–mass spectrometry (GC/MS). Finally, all produced wines were evaluated by quantitative descriptive analysis by two groups of experts; the Greek team of oenologists from Santorini Island specialized in Assyrtiko wines, and the French team of oenologists specialized in wine from Bordeaux. As expected, the inoculated strain was the one that dominated the fermentation process, but nine *S. cerevisiae* indigenous strains were also identified in the produced wines. *Lachancea thermotolerans* produced 1 g/L of lactic and also modulated the volatile profile of the wines independently of the *S. cerevisiae* strain used. The origin of the panelists played an important role in bringing up sensorial traits, such as acidity. Our results led to a new interesting application of *L. thermotolerans* for white wine production adapted to climate change claims.

## 1. Introduction

The use of non-*Saccharomyces* yeasts in winemaking has been extensively assessed, as they may confer a series of advantages. Indeed, the modification of sensorial complexity, as well as the reduction of ethanol and biogenic amine content, through co-fermentation of *Saccharomyces cerevisiae* with non-*Saccharomyces* yeasts such as *Hanseniaspora vineae*, *H. uvarum*, *Lachancea thermotolerans*, *Metschnikowia pulcherrima*, *Pichia fermentans*, *Starmerella bacillaris*, *Torulaspora delbrueckii*, and *Wickerhamomyces anomalus* has been reported [1,2,3,4,5,6,7,8,9,10,11,12,13,14,15,16,17]. In addition, their capacity to act as bioprotective cultures, and therefore lead to a reduction of SO_2_ addition or even confer probiotic benefits to the consumer, have also been considered [18,19,20,21,22,23,24,25]. As a result, a series of non-*Saccharomyces* yeasts are currently commercially available.

Among the non-*Saccharomyces* yeasts studied, *L. thermotolerans* constitutes a very interesting alternative, as it may contribute to addressing the negative effects of climate change in winemaking. More specifically, global warming resulted in grape ripening acceleration, which in turn allowed for grape musts with lower acidity and higher carbo-hydrate concentration [26]; the first compromises wine stability, while the second results in wines with higher ethanol content. Both constitute significant problems for the wine industry [27]; therefore, the quest for tackling strategies is ongoing. *Lachancea thermotolerans* may provide a feasible solution, as it has the ability to produce lactic acid through carbohydrate catabolism, increasing the acidity of the must and depriving carbon sources for ethanol production by *S. cerevisiae* [28]. Indeed, the fermentation by *L. thermotolerans* and *S. cerevisiae* of grape musts of many varieties, including Airen, Albarino, Babic, Blatina, Cabernet Sauvignon, Emir, Frankovka, Garganega, Mencia, Merlot, Muscat, Pinot Blanc, Plavac Mali, Riesling, Sangiovese, Sauvignon Blanc, Shiraz, Treixadura, and Trnjak, has highlighted the feasibility of this strategy, as well as the modification of sensorial perception of the wines produced [29,30,31,32,33,34,35,36,37,38,39,40,41,42,43].

The *Vitis vinifera* cv. Assyrtiko is indigenous to the island of Santorini, specifically adapted to its unique edaphoclimatic conditions. The PDO Santorini made by this variety is characterized by a dense structure, crisp acidity, and minerality. This distinctive character is at risk due to global warming. Indeed, a decrease in acidity and an increase in ethanol content would be detrimental to the unique sensorial qualities of PDO Santorini. Therefore, the need for approaches capable of addressing this issue is imperative. The aim of the present study was to sequentially ferment grape must of the Assyrtiko variety by *L. thermotolerans* and three *S. cerevisiae* strains and evaluate the effect on physicochemical parameters and sensorial perception. Especially regarding the latter, sensory evaluation was performed by two expert panels, one familiarized with the specific organoleptic features of this variety and one that was not accustomed to them.

## 2. Materials and Methods

### 2.1. Microbial Strains and Culture Conditions

Two strains under industrial development, namely *Lachancea thermotolerans* Lt1 and *Saccharomyces cerevisiae* Sc1, and two *S. cerevisiae* strains previously isolated from the Greek terroir, namely A26Y23 and A6Y10 [44], were used throughout this study. The strains were stored at −20 °C in Nutrient Broth supplemented with 20% glycerol. Before experimental use, the strains were grown twice in YM broth (1% glucose, 0.5% peptone, 0.3% yeast extract, 0.3% malt extract) at 25 °C for 48 h.

### 2.2. Experimental Design and Winemaking Conditions

Grapes of the Assyrtiko variety, grown in Santorini, were harvested, manually destemmed, and crushed, followed by the addition of sodium metabisulfite (50 mg/L) (Scharlab S.A, Barcelona, Spain). After cold clarification and the addition of Lallzyme C-Max, (0.5 g/hL) (Lallemand, Montreal, QC, Canada), the must was decanted into twelve stainless steel tanks of 1 ton each. Then, the must (Bé: 13.2, density: 1.0963, and pH: 3.1) was inoculated according to the cases shown in Table 1. In the first three cases, the *L. thermotolerans* strain was inoculated in the form of biomass paste at approximately 10^6^ CFU/mL. After 48 h, lactic acid was measured and each of the *S. cerevisiae* strains was inoculated, also in the form of biomass paste, at 10^6^ CFU/mL. In cases W4-W6, only each of the *S. cerevisiae* strains were inoculated in the must. Case W6 can be considered as the control, since this strain has been used for wine production by wineries. Forty-eight hours after the inoculation, the addition of 300 mg/L NUTRISTART™ (Laffort, Floirac, France) took place. Fermentations were carried out at 18 °C. The fermentation was considered to be complete when the carbohydrates (glucose and fructose) were depleted (less than 2 g/L).

### 2.3. Chemical Analyses

#### 2.3.1. Standard Oenological Parameters

Fermentation was monitored at daily intervals through the measurement of the residual glucose, fructose, and alcohol content. The former was performed enzymatically [45], while the latter used NIR spectrometry [46]. In addition, the pH value, total and volatile acidity, L-lactic acid, L-malic acid, and total and free SO_2_ were determined in the musts immediately upon crushing of the grapes, as well as after the fermentation was completed. These analyses were performed through enzymatic kits adapted for a Y15 BioSystems auto-analyzer (BioSystems, Barcelona, Spain).

#### 2.3.2. Identification and Quantification of Volatile Compounds

Quantification of higher alcohols, esters, and terpenoids was performed by headspace solid-phase microextraction (HS-SPME) coupled with gas chromatography-mass spectrometry (GC-MS). The analysis took place according to Dimopoulou et al. [47]. In brief, the sample (25 mL) was placed in a 40 mL vial, along with 25 μg of 3-octanol (1 g/L) (internal standard), 3 g NaCl, and a magnetic stir bar. The vial was sealed with a silicon septum containing a screw-top cap and placed on a hotplate magnetic stirrer. Equilibration took place through stirring at 750 rpm at 40 °C for 10 min. The volatile compounds were absorbed on a DVB/CAR/PDMS 75 μm fiber at 40 °C for 30 min, and the fiber was subsequently inserted into the injector of an Agilent 7890A GC (Santa Clara, CA, USA) equipped with an Agilent 5873C MS detector. The injection mode was splitless. Separation of the volatile compounds took place in a DBWAX capillary column (30 m × 0.25 mm i.d., 0.25 μm film thickness) using helium as a gas carrier at a flow rate of 1.2 mL/min. Injector and MS-transfer line temperatures were 250 °C and 260 °C, respectively. The initial oven temperature was 30 °C for 5 min and was subsequently raised to 220 °C at 4 °C/min and maintained for 20 min. Thiols were determined according to the method described by Tominaga et al. [48]. Identification and quantification of the volatile compounds took place through the NIST library and commercial standards with external calibration curves.

### 2.4. Microbiological Analyses and Molecular Typing

Microbiological analyses were performed at the end of each fermentation, when approximately 2/3 of sugars were depleted. The population of the yeasts belonging to the *Saccharomyces* genus was enumerated through plating serial dilutions on Wallerstein Laboratory Nutrient agar (WLN) and incubating at 28 °C for 48 h. Lysine medium agar and incubation at 28 °C for 48–72 h was used for enumeration of the non-*Saccharomyces* yeast population. All colonies present in the final dilution of each medium were purified by successive subculturing under the same conditions and subjected to DNA extraction and verification of their identity through interdelta sequence profile analysis, according to Tzamourani et al. [44].

### 2.5. Sensory Analysis

The wines produced were subjected to descriptive analysis [49] by two expert panels. Panel A consisted of 13 trained judges (9 females and 5 males aged from 30 to 65), residing in Bordeaux, France. These judges were considered to be unfamiliar with the organoleptic perception of wines from the Assyrtiko cultivar. On the other hand, panel B consisted of 11 trained judges (3 females and 9 males aged from 27 to 64) residing on the island of Santorini, and therefore very familiar with wines produced by the Assyrtiko cultivar. The judges were asked to assess the following descriptors: 1. Olfactory (aroma intensity, complexity, floral, citrus, lactic, vegetal, yellow fruits); 2. Gustatory (aftertaste, acidity, balance, bitterness, sweetness, mouthfeel); and 3. Overall preference. Sensory analysis was performed according to Dimopoulou et al. [47] and evaluation took place using a 10-point scale (1: absence; 10: very strong). 

### 2.6. Statistical Analysis

The fermentations were carried out in duplicate. Statistically significant differences between the attributes assessed were evaluated by one and two-way Analysis of Variance (ANOVA), followed by Tukey’s post hoc test, performed using JMP version 3.1.5 software (SAS Institute Inc., Cary, NC, USA). Principal component analysis (PCA) was employed to indicate relationships between variables and samples, and it was performed using R version 3.6.2.

## 3. Results

Lactic acid was detected 48 h after *L. thermotolerans* was inoculated, indicating the survival of the non-*Saccharomyces* species at the beginning of fermentation. After the inoculation of *S. cerevisiae* strains, the production of lactic acid ceased, and no non-*Saccharomyces* yeasts were detected. The moment that *S. cerevisiae* was added (48 h), the densities of the fermented musts were W1: 1.0935, W2: 1.0935, W3: 1.0935, W4: 1.0823, W5: 1.089, and W6: 1.078, and the pH value was 3.1.

**Figure 1:**
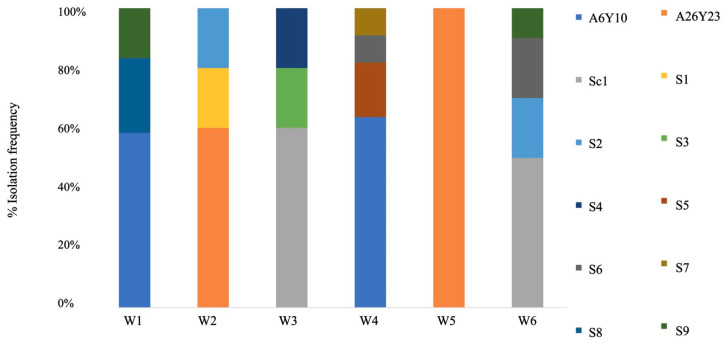
The microecosystem composition at the end of each fermentation (inoculation cases: W1: *L. thermotolerans* strain Lt1—*S. cerevisiae* strain A6Y10; W2: *L. thermotolerans* strain Lt1—*S. cerevisiae* strain A26Y23; W3: *L. thermotolerans* strain Lt1—*S. cerevisiae* strain Sc1; W4: *S. cerevisiae* strain A6Y10; W5: *S. cerevisiae* strain A26Y23; W6: *S. cerevisiae* strain Sc1). The indigenous *S. cerevisiae* strains are indicated by the letter S (S1–S9).

The microbiological analyses performed on the final day of each fermentation revealed that the *Saccharomyces* population ranged between 6.3–7.2 log CFU/mL, while the non-*Saccharomyces* one was below the enumeration limit (2 log CFU/mL). A total of 200 colonies were obtained from the plates of the final dilution, as described in paragraph 2.4. Interdelta sequence profile analysis suggested that the *S. cerevisiae* strains used as inoculum, either in mono- or co-culture with the *L. thermotolerans* strain, dominated their respective yeast microcommunity (Figure 1). Indeed, *S. cerevisiae* strain A6Y10 dominated cases W1 and W4, *S. cerevisiae* strain A26Y23 cases W2 and W5, and *S. cerevisiae* strain Sc1 cases W3 and W6. The *L. thermotolerans* strain was not detected at all. Apart from the *S. cerevisiae* strains used as inocula, nine other strains, designated S1–S9, were also recovered from the Wallerstein Laboratory Nutrient agar that was employed for the enumeration of the *Saccharomyces* population.

In Table 2, the classical oenological characteristics of the wines produced in the present study are shown. Assyrtiko must fermentation was completed after 12 d when the *L. thermotolerans* strain was used (cases W1, W2, and W3).

This constitutes a significant acceleration, compared to the 18 d that the *S. cerevisiae* strains A6Y10 and A26Y23 required to complete the fermentation when used as monocultures (cases W4 and W5). On the contrary, the *L. thermotolerans* strain seemed to decelerate the fermentation driven by *S. cerevisiae* strain Sc1, which was completed after 8 d, when the latter was used as a monoculture (case W6). The final alcoholic volume ranged between 13.5–13.8% *v/v*, with *S. cerevisiae* strain Sc1 resulting in lowest and *S. cerevisiae* strains A6Y10 and A26Y23 in the highest ones. Regarding total and volatile acidity, the use of the *L. thermotolerans* strain resulted in alleviating the differences between the *S. cerevisiae* strains. More specifically, the lowest total acidity of 6.48 g/L was obtained by *S. cerevisiae* strains A6Y10 (case W4) and Sc1 (case W6). On the other hand, the highest total acidity of 6.9 g/L was obtained by *S. cerevisiae* strain A26Y23 (case W5). The use of *L. thermotolerans* resulted in total acidity ranging from 7.14 to 7.26 g/L, without statistically significant differences between the cases W1–W3. Similarly, the lowest volatile acidity of 0.2 g/L was obtained by *S. cerevisiae* strain Sc1, whereas the volatile acidity of the wines made by *S. cerevisiae* strains A6Y10 and A26Y23 was 0.6 and 0.61 g/L, respectively. The wines made with the use of the *L. thermotolerans* strain exhibited volatile acidity ranging from 0.41 to 0.43 g/L. This uniformity was also observed in the case of the pH value, as well as in the final L-lactic and L-malic acid concentrations. The pH value of all the wines presented no statistically significant differences and ranged between 2.9–3.0. No L-lactic acid was detected in the wines made by the monocultures of the *S. cerevisiae* strains, while L-malic acid ranged within 1.1–1.3 g/L. On the contrary, 1.0–1.1 g/L L-lactic acid and 1.0 g/L L-malic acid were detected in the wines made with the use of the *L. thermotolerans* strain. The total and free SO_2_ ranged between 87 and 117 mg/L and between 17 and 28 mg/L, respectively. In the case of total SO_2_, the lowest concentration was observed in case W1 and the highest in case W4. In the case of free SO_2_, the lowest concentration was observed in cases W1, W3, and W5 and the highest in case W6.

**Figure 2:**
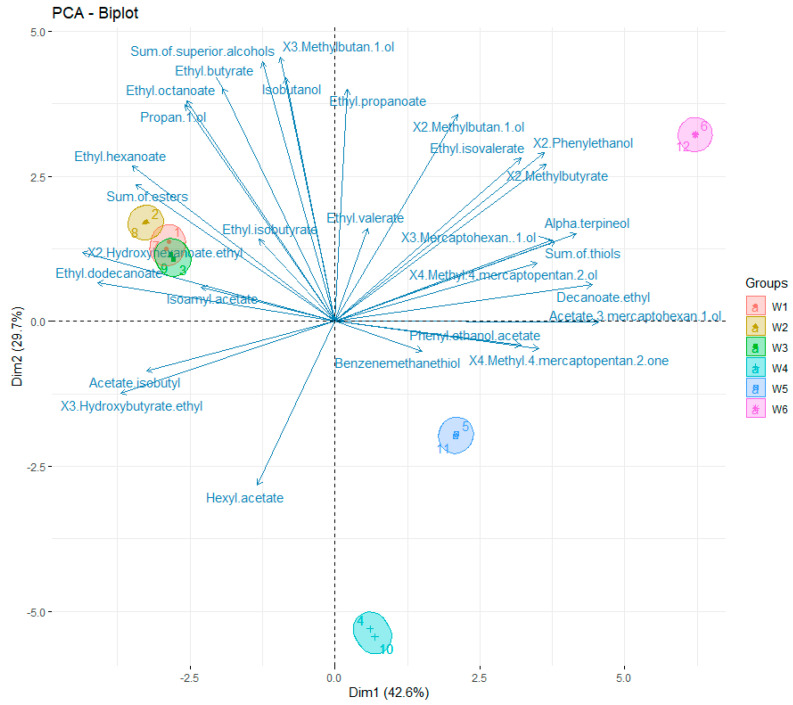
Principal component analysis of 27 volatile compounds of Assyrtiko wines fermented with monocultures of *S. cerevisiae* strains A6Y10, A6Y23, and Sc1 and their co-cultures with *L. thermotolerans* strain Lt1. W1: *L. thermotolerans* strain Lt1—*S. cerevisiae* strain A6Y10; W2: *L. thermotolerans* strain Lt1—*S. cerevisiae* strain A26Y23; W3: *L. thermotolerans* strain Lt1—*S. cerevisiae* strain Sc1; W4: *S. cerevisiae* strain A6Y10; W5: *S. cerevisiae* strain A26Y23; W6: *S. cerevisiae* strain Sc1.

In Table 3, the volatile compounds quantified in the Assyrtiko wines made under the different inoculation cases assessed are exhibited. The use of different *S. cerevisiae* strains resulted in the production of wines with statistically significant differences in their volatile compounds content. Utilization of the *L. thermotolerans* strain resulted in their quantitative modification, at least in the majority of the cases. A total of four higher alcohols, namely 2-methylbutan-1-ol, 3-methylbutan-1-ol, isobutanol, and propan-1-ol, were detected and quantified in the wines produced. The wine made by *S. cerevisiae* strain A6Y10 (case W4) had the lowest amount of each higher alcohol. On the contrary, the highest amounts were found in cases W2 and W6. The effect that the utilization of the *L. thermotolerans* strain had on higher alcohol production seemed to be strain-dependent. More specifically, combination with strain A6Y10 resulted in the increase of the concentration of all higher alcohols produced. Similarly, combination with strain A26Y23 resulted in the increase of the concentration of all higher alcohols except for 2-methylbutan-1-ol, the concentration of which was decreased. Finally, combination with *S. cerevisiae* strain Sc1 resulted in a mixed response, namely, the increase of propan-1-ol concentration, the decrease of 2-methylbutan-1-ol and 3-methylbutan-1-ol concentration, and no change in the concentration of isobutanol. A total of five thiols were detected and quantified in the majority of the wines produced. The highest amounts were mostly produced in the wines made by *S. cerevisiae* monocultures, particularly strains A26Y23 and Sc1. The only exception was benzenemethanethiol, the highest amount of which was produced when the *L. thermotolerans* strain was combined with strain A6Y10. The utilization of the *L. thermotolerans* strain resulted in the reduction of the 4-methyl-4-mercaptopentan-2-one and acetate 3-mercaptohexan-1-ol concentration and the increase of benzenemethanethiol concentration, regardless of the *S. cerevisiae* strain employed. On the contrary, the effect on 3-mercaptohexan-1-ol and 4-methyl-4-mercaptopentan-2-ol concentration seemed to be strain-dependent. Indeed, the combination of the *L. thermotolerans* strain with *S. cerevisiae* strains A6Y10, Sc1, and A26Y23 resulted in a decrease of 3-mercaptohexan-1-ol concentration in the first two and an increase in the last one. Similarly, 4-methyl-4-mercaptopentan-2-ol concentration was reduced upon combination with the *L. thermotolerans* strain with *S. cerevisiae* strains A26Y23 and Sc1 and remained unchanged upon combination with strain A6Y10. A total of 13 ethyl esters were detected and quantified in the majority of the wines produced. The majority of the highest and lowest amounts were found in the wines made by *S. cerevisae* strains Sc1 and A6Y10, respectively. The effect of *L. thermotolerans* utilization on the ethyl esters produced seemed to be strain-dependent. Indeed, only in the cases of ethyl 2-methylbutyrate, ethyl octanoate, ethyl 2-hydroxyhexanoate, ethyl dodecanoate, and ethyl decanoate was the same response recorded. More specifically, the concentration of ethyl 2-methylbutyrate remained unchanged, the concentration of ethyl decanoate decreased, and the concentration of ethyl octanoate, ethyl 2-hydroxyhexanoate, and ethyl dodecanoate increased when the *L. thermotolerans* strain was employed. Regarding the remaining ethyl esters, although the response recorded was not common, in the majority of the cases, an increase in their concentration was recorded upon utilization of the *L. thermotolerans* strain. A total of four acetate esters were detected and quantified in the wines produced. The highest amounts were produced by *S. cerevisiae* strain A26Y23, while most of the lowest concentrations were produced by *S. cerevisiae* strain Sc1. Utilization of the *L. thermotolerans* strain had a strain-dependent effect on the acetate esters produced. Interestingly, the concentration of all acetate esters produced by *S. cerevisiae* strain A26Y23 was reduced when it was combined with the *L. thermotolerans* strain. On the other hand, the concentration of all acetate esters produced by *S. cerevisiae* strain Sc1, other than phenylethyl acetate, increased when it was combined with the *L. thermotolerans* strain. The only terpenoid compound quantified was α-terpineol. The highest concentration was observed in the wine made by *S. cerevisiae* strain Sc1, while the lowest was recorded when this strain was combined with the *L. thermotolerans* strain. In general, the effect of *L. thermotolerans* utilization on α-terpineol concentration seemed to be strain-dependent. Collectively, the lessening of the differences between the W4, W5, and W6 cases, which was observed in the classical oenological parameters when *L. thermotolerans* was employed, was also evident in the case of the volatile compounds. Indeed, as shown in Figure 2, cases W4, W5, and W6, i.e., the wines made by the monocultures of *S. cerevisiae* strains A6Y10, A6Y23, and Sc1, respectively, were not grouped together. On the contrary, when the *L. thermotolerans* strain was employed, all cases (W1, W2, and W3) were grouped together.

**Figure 3:**
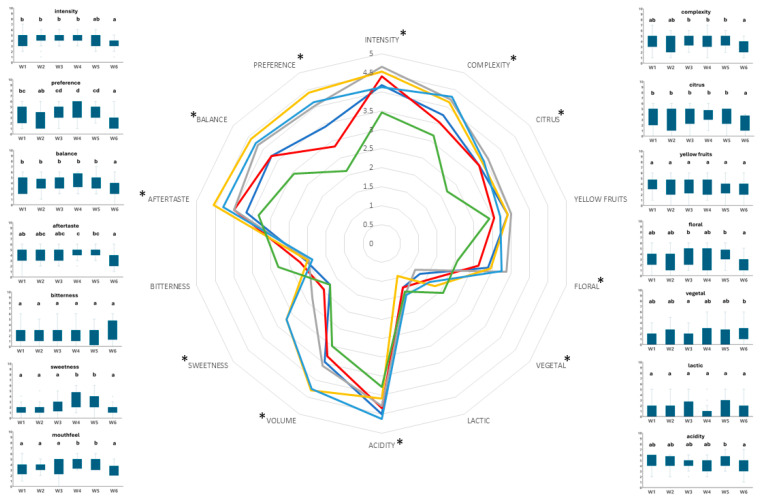
Sensory description of the wines produced in the present study. W1: *L. thermotolerans* strain Lt1—*S. cerevisiae* strain A6Y10 (dark blue); W2: *L. thermotolerans* strain Lt1—*S. cerevisiae* strain A26Y23 (red); W3: *L. thermotolerans* strain Lt1—*S. cerevisiae* strain Sc1 (gray); W4: *S. cerevisiae* strain A6Y10 (yellow); W5: *S. cerevisiae* strain A26Y23 (light blue); W6: *S. cerevisiae* strain Sc1 (green). The asterisk next to each attribute indicates statistically significant differences between the different wines. These are presented by the box and whisker charts. Different letters above each box designate statistically significant differences (*p* < 0.05) of the specific attribute between the different wines.

In Figure 3, the descriptive analysis of the sensorial qualities of the wines produced is presented. In general, the descriptors ‘lactic’, ‘yellow fruit’, and ‘bitterness’ were the ones in which no statistically significant differences between the wines were observed. Comparison of the wines made by *S. cerevisiae* monocultures, namely, cases W4, W5, and W6, revealed no statistically significant differences regarding the descriptor ‘vegetal’. Regarding the remaining descriptors, the lowest evaluation was received by the wine made by *S. cerevisiae* strain Sc1. The utilization of the *L. thermotolerans* strain resulted in the statistically significant improvement of the degrees received by the wine made by *S. cerevisiae* strain Sc1 for the descriptors ‘intensity’, ‘complexity’, ‘citrus’, ‘floral’, ‘vegetal’, ‘balance’, and ‘preference’. On the other hand, utilization of the *L. thermotolerans* strain resulted in a statistically significant reduction of the degrees received by the wines made by the other two *S. cerevisiae* strains, regarding the descriptors ‘mouthfeel’, ‘sweetness’, and ‘preference’. In addition, the wine made by the coculture of the *L. thermotolerans* strain and the *S. cerevisiae* strain A6Y10 (case W1) presented a statistically significant reduction of the descriptor ‘aftertaste’, compared to the wine made only by the *S. cerevisiae* strain A6Y10 (case W4). Overall, the most preferred wines were the ones made by the monoculture of *S. cerevisiae* strains A6Y10 (case W4) and A26Y23 (case W5) and the one made by the coculture of the *L. thermotolerans* strain and the *S. cerevisiae* strain Sc1 (W3).

In Figure S1, the sensory description of the wines produced in the present study, as well as the evaluation of each descriptor by the French and Greek panels, is presented. In general, disagreement between the two panels was mostly detected in wines W1 and W2. Indeed, out of a total of 14 descriptors, statistically significant differences were observed in 7 and 10. On the other hand, a general agreement between the two panels was observed in the rest of the cases, as statistically significant differences were detected in less than 5 descriptors each. The two teams of evaluators were in total agreement only in the case of the descriptor ‘yellow fruits’, as no statistically significant differences between the panels were observed. On the contrary, the descriptor ‘vegetal’ was the only one in which statistically significant differences between the two panels were observed in all examined wines. In addition, the differences regarding wines W3, W4, and W5 were at *p* < 0.001. Similarly, the two panels had different perceptions regarding the descriptor ‘citrus’ in all wines, except for W5. Furthermore, in the case of wines W1, W2, and W3, the differences were at *p* < 0.001. The different evaluations of the descriptors between the panels revealed no particular trend, with the exception of the descriptor ‘acidity’, in which statistically significant differences between the panels were observed in wines W1, W4, W5, and W6. In all cases, the Greek panel described these wines as being more acidic, than did the French panel.

## 4. Discussion

Climate change is a threat to grapevine growers and the wine industry. Indeed, a variety of bioclimatic indices have been developed in order to scrutinize productivity in the field, accompanied by possible adaptation strategies [50,51,52,53,54,55,56,57,58,59,60]. At the winemaking level, the proposed strategies include chemical and microbiological acidification [61]. Chemical acidification through the use of citric, tartaric, or malic acids is not a feasible option due to their instability and the effect on sensorial properties. On the contrary, lactic acid is more stable and organoleptically softer than the aforementioned acids [62]. Therefore, biological acidification through the use of *L. thermotolerans*, with its unique metabolic attributes, in combination with *S. cerevisiae*, is promoted as a feasible option [63]. In our study, we would like to go further and examine this acidification effect in an already acidic variety, in terms of microbial dominance and sensory attributes.

Time of inoculation, inoculum ratio, and fermentation temperature have been reported to affect the growth and persistence of *L. thermotolerans* and *S. cerevisiae* [64,65,66]. Sequential fermentation seems to be the strategy of choice in order to avoid co-existence with *S. cerevisiae*. According to this scheme, *L. thermotolerans* is inoculated first, and when the acidity reaches the desired level, depending on the capacity of the strain, inoculation with *S. cerevisiae* takes place. This way, acidification takes place and *L. thermotolerans* experiences no antagonism by *S. cerevisiae*. On the contrary, co-inoculation will result in antagonism for nutrients, especially nitrogen, and redirection of the *L. thermotolerans* carbon catabolism from glycolysis to the pentose phosphate pathway; both will result in the reduction of lactic acid production [28,67]. Concerning *S. cerevisiae* implantation control, besides the strain A26Y23, which could dominate at 100% (W5), all the other inoculated strains were present at approximately 60%. As Assyrtiko must is known for its high acidity and low pH, the indigenous strains are well adapted and are able to survive until the end of the fermentation process [47]. This statement is in accordance with the implantation control results, where the indigenous strains remained in an increased population until the end of alcoholic fermentation.

In general, low fermentation temperatures (≤20 °C) enhance the competition potential of *L. thermotolerans*, especially when *S. cerevisiae* is sequentially and not simultaneously inoculated, leading to protracted persistence in high populations [68]. However, this is observed in laboratory fermentations using pasteurized grape juice and not under industrial fermentation conditions. In the latter case, *L. thermotolerans* seems to lack effective competitiveness and therefore its final biomass is rather restricted [68]. In the present study, in which fermentation took place under industrial conditions, *S. cerevisiae* strains were added 48 h after *L. thermotolerans.* Based on the oenological analysis, this period was adequate for the activation of lactic acid production. 

Regarding the duration of fermentation, it has been stated that incorporation of *L. thermotolerans* results in prolongation for a few days [28,63,69]. This was also the case in the present study, but only as far as the fermentation by *S. cerevisiae* strain Sc1 was concerned. In the case of *S. cerevisiae* strains A6Y10 and A26Y23, fermentation was accelerated. Such an effect could be attributed to the production of metabolites by the *L. thermotolerans* strain employed that favor the development of the specific *S. cerevisiae* strains. Since this effect is strain-dependent, further study is necessary in order to identify and elucidate it. These changes in the duration of fermentation that were observed in the present study were not accompanied by changes in the total alcoholic volume (TAV). Indeed, no statistically significant differences were observed due to the addition of *L. thermotolerans*. This concurs with the results presented by Blanco et al. [34], Romani et al. [35], Snyder et al. [39], Ženišová et al. [40], Vaquero et al. [41], and Mucalo et al. [43]. On the contrary, reduction of TAV due to the use of *L. thermotolerans* was reported by Benito et al. [29], Castrillo et al. [32], Morata et al. [33], Fairbairn et al. [36], Korenika et al. [38], and in some experimental cases studied by Hranilovic et al. [37] and Gallo et al. [42]. These differences can be attributed to the different metabolic capacities of the *L. thermotolerans* and *S. cerevisiae* strains employed in each study, as well as the hostile environment of Assyrtiko wine.

The aim of the present work was to study how changing the composition of acids, through *L. thermotolerans* utilization, could affect wine sensorial and oenological characteristics. According to our results, *L. thermotolerans* succeeded to produce lactic acid during the first 48 h of fermentation, even in highly acidic must, resulting in enhancing total acidity at an average of 9%, abolishing the statistically significant differences that were observed in the wines made only by the *S. cerevisiae* strains. Similarly, the statistically significant differences observed in the wines made only by the *S. cerevisiae* strains regarding volatile acidity were also eliminated. Indeed, when *L. thermotolerans* was utilized, the volatile acidity was increased by approximately 50%, compared to the wines made only by *S. cerevisiae* strains A6Y10 and A26Y23, but was reduced by the same percentage, compared to the wine made only by strain Sc1. When *L. thermotolerans* is used, an increase in total acidity, whether accompanied or not by an increase in volatile acidity, always correlates to the production of lactic acid and is very frequently reported [30,31,33,37,38,39,40,41,42,43]. There are also cases in which no statistically significant differences in total and volatile acidity were reported when the lactic acid production was low [32,34,35]. However, this is the first time that a decrease in volatile acidity through the use of *L. thermotolerans* has been reported. As in the case of the effect of the *L. thermotolerans* strain used in the present study on fermentation duration, this effect is strain-dependent, indicating that the trophic relationships between the strains of the two species are complicated and deserve further attention. Additionally, the low pH of Assyrtiko must may induce this observed production of acetic acid by the yeast in order to succeed in its adaptation.

The aforementioned changes in total and volatile acidity observed in the present study were not accompanied by a statistically significant decrease in pH value. This is in accordance with the results presented by many authors [29,32,34,35,40,43]. However, there are also many studies employing *L. thermotolerans* that report a statistically significant decrease in the wine’s pH under high lactic acid productions [33,36,37,38,39,41,42].

Another interesting trait of *L. thermotolerans* is malic acid consumption; the majority of the strains have been reported to consume 10–20% of it, while consumption that may exceed 50% has also been reported [28,29,33,34,35,37,38,39]. In the present study, malic acid consumption ranged from 9 to 23%, concurring with the aforementioned results. However, since this is a strain-dependent property, some studies reported no statistically significant reduction of malic acid [32,34].

Several *L. thermotolerans* and *S. cerevisiae* strains have been sequentially employed to ferment musts from a wide variety of grape cultivars. In the majority of the cases, incorporation of the *L. thermotolerans* strain resulted in an increase in the production of isobutanol and a decrease in the production of ethyl hexanoate, ethyl octanoate, ethyl decanoate, isoamyl acetate, and hexyl acetate [29,32,33,34,37,38,40,41,42,43]. As far as the rest of the volatile compounds were concerned, no particular trend could be identified in the available literature, indicating the profound effect of the must composition and the metabolic capacity of the yeast strains employed. Unfortunately, no reports are available regarding the effect of *L. thermotolerans* on the production of thiols; therefore, the reduction of their concentration, which was observed in the present study, cannot be compared to other studies. 

Assyrtiko wines from Santorini are characterized by sharp acidities with great aging potential. In our study, we would like to find out how changing the composition of acids in wine may impact its sensorial properties. Two panels of experts were chosen for the study; the first was a group of enologists from the region of Bordeaux in France, representing the wine experts who are not familiar with wines from Assyrtiko; the second was a group of winemakers from the region of Santorini in Greece, experts that were familiar with the variety of the volcanic terroir. Based on our results, the increase of total acidity provoked by *L. thermotolerans* was not noticed by any of the two panels of experts, showing that 1 g/L of lactic acid does not have a significant impact on lactic or acidity perception. This result is in accordance with a previous study on the role of compound interactions on red wine taste perception, where a concentration of more than 1.4 g/L of lactic acid can contribute to a sour taste of wine [70]. Nevertheless, the expert panel of Santorini seemed to discriminate the acid sensation based on acid composition and not on total acidity, as they found the wines with less malic acid and more lactic acid to be less acidic. Recently, it has been shown that the sour taste related to the acidic attribute is mostly correlated to individual chemical compounds of the wine and not so related to the titratable acidity [71]. Additionally, the perceived acidity of wine was statistically enhanced by volatile compounds. In fact, the authors suggested cognitive interaction with taste perception, as the acids in the volatile reconstitution of model wine were unlikely to have contributed significantly to the perception of acidity [72]. As the Assyrtiko of Santorini is considered a low-intensity aromatic wine characterized by minerality, the experts of Santorini could possibly correlate the unusual, enhanced aromatic intensity of the experimental wines caused by the chosen yeast to increased acidity, indicating that the perception of acidity is a complex phenomenon that goes further than the concentration of acids.

## 5. Conclusions

Acidification of wines during alcoholic fermentation through selected yeast strains is a feasible solution in winemaking to face global warming. This phenomenon can be achieved by sequential fermentation of *L. thermotolerans* and *S. cerevisiae* strains, even in highly acidic wines, such as the Greek Assyrtiko. The outcome of the sensorial attributes is highly dependent on the yeast strain used, the final composition of acids, and the expertise of the panel.

## Figures and Tables

**Table 1:** Inoculation cases assessed in the present study.

Code	Inoculum
W1	*L. thermotolerans* strain Lt1—*S. cerevisiae* strain A6Y10
W2	*L. thermotolerans* strain Lt1—*S. cerevisiae* strain A26Y23
W3	*L. thermotolerans* strain Lt1—*S. cerevisiae* strain Sc1
W4	*S. cerevisiae* strain A6Y10
W5	*S. cerevisiae* strain A26Y23
W6	*S. cerevisiae* strain Sc1

**Table 2:** Classical oenological parameters of the wines produced.

	W1	W2	W3	W4	W5	W6
Fermentation duration (d)	12 (0)	12 (0)	12 (0)	18 (0)	18 (0)	8 (0)
TAV (% *v/v*)	13.7 (0.1) ^ab^	13.7 (0.2) ^ab^	13.6 (0.2) ^ab^	13.8 (0.1) ^b^	13.8 (0.1) ^b^	13.5 (0.2) ^a^
Glucose + Fructose (g/L)	0.7 (0.01) ^c^	0.7 (0.03) ^c^	0.8 (0.04) ^d^	1.7 (0.01) ^e^	0.4 (0.04) ^a^	0.5 (0.03) ^b^
Total acidity (g/L)	7.26 (0.14) ^c^	7.14 (0.21) ^b c^	7.23 (0.32) ^c^	6.48 (0.16) ^a^	6.90 (0.12) ^b^	6.48 (0.22) ^a^
Volatile acidity (g/L)	0.43 (0.01) ^b^	0.41 (0.01) ^b^	0.41 (0.01) ^b^	0.60 (0.03) ^c^	0.61 (0.02) ^c^	0.20 (0.02) ^a^
pH	2.9 (0.01) ^a^	2.9 (0.05) ^a^	2.9 (0.07) ^a^	3.0 (0.04) ^a^	3.0 (0.08) ^a^	2.9 (0.09) ^a^
L-lactic acid (g/L)	1.0 (0.04) ^b^	1.0 (0.06) ^b^	1.1 (0.02) ^c^	0 (0.0) ^a^	0 (0.0) ^a^	0 (0.0) ^a^
L-malic acid (g/L)	1.0 (0.05) ^a^	1.0 (0.03) ^a^	1.0 (0.02) ^a^	1.2 (0.05) ^c^	1.3 (0.02) ^d^	1.1 (0.05) ^b^
Total SO_2_ (mg/L)	87 (3.1) ^a^	99 (4.7) ^b^	103 (6.8) ^b^	117 (5.5) ^c^	107 (6.7) ^b^	105 (4.9) ^b^
Free SO_2_ (mg/L)	17 (1.2) ^a^	23 (2.1) ^bc^	22 (2.2) ^ab^	28 (4.6) ^cd^	22 (3.5) ^ab^	31 (3.3) ^d^

The average values are presented. Standard deviation is given in parentheses. Different letters in each row designate statistically significant differences (*p* < 0.05). W1: *L. thermotolerans* strain Lt1—*S. cerevisiae* strain A6Y10; W2: *L. thermotolerans* strain Lt1—*S. cerevisiae* strain A26Y23; W3: *L. thermotolerans* strain Lt1—*S. cerevisiae* strain Sc1; W4: *S. cerevisiae* strain A6Y10; W5: *S. cerevisiae* strain A26Y23; W6: *S. cerevisiae* strain Sc1.

**Table 3:** Volatile compounds of the Assyrtiko wines made under the different inoculation cases examined.

	W1	W2	W3	W4	W5	W6
Higher alcohols (mg/L)						
2-Methylbutan-1-ol	25.7 (0.42) ^b^	25.2 (0.49) ^b^	24.9 (0.21) ^b^	15.1 (0.14) ^a^	29.6 (0.28) ^c^	33.5 (0.21) ^d^
3-Methylbutan-1-ol	221 (1.4) ^cd^	242 (7.6) ^e^	218 (2.7) ^c^	107 (0.6) ^a^	152 (2.7) ^b^	228 (4.1) ^d^
Isobutanol	29.7 (0.49) ^b^	32.6 (0.21) ^c^	33.0 (0.49) ^c^	22.2 (0.42) ^a^	21.75 (0.48) ^a^	33.1 (0.43) ^c^
Propan-1-ol	81.1 (0.63) ^d^	92.8 (0.64) ^e^	82.8 (1.48) ^d^	23.0 (0.63) ^a^	55.3 (0.62) ^b^	60.9 (0.42) ^c^
Thiols (ng/L)						
4-methyl-4-mercaptopentan-2-one	0 (0.0) ^a^	3.05 (0.212) ^b^	3.00 (0.141) ^b^	3.30 (0.141) ^b^	6.95 (0.212) ^d^	6.05 (0.221) ^c^
3-mercaptohexan-1-ol	704 (2.6) ^c^	676 (8.3) ^b^	674 (7.1) ^b^	1245 (10.2) ^d^	500 (3.2) ^a^	2755 (20.0) ^e^
Acetate 3-mercaptohexan-1-ol	56.3 (1.62) ^a^	58.7 (0.56) ^a^	66.0 (0.99) ^d^	82.8 (1.20) ^b^	78.5 (1.34) ^c^	108 (3.3) ^e^
4-methyl-4-mercaptopentan-2-ol	18.8 (0.28) ^a^	20.8 (1.10) ^b^	21.8 (0.49) ^b^	17.8 (0.63) ^a^	32.1 (1.06) ^d^	29.1 (0.77) ^c^
Benzenemethanethiol	2.20 (0.141) ^e^	0 (0.0) ^a^	0.475 (0.035) ^b^	1.18 (0.021) ^cd^	1.08 (0.169) ^c^	1.35 (0.063) ^d^
Ethyl esters (mg/L)						
Ethyl propanoate	333 (5.6) ^c^	289 (14.8) ^b^	288 (18.4) ^b^	182 (17.7) ^a^	282 (9.2) ^b^	329 (7.1) ^c^
Ethyl isobutyrate	109 (5.6) ^c^	105 (4.9) ^bc^	97.5 (2.12) ^b^	104 (2.1) ^bc^	43.5 (4.94) ^a^	104 (1.4) ^bc^
Ethyl butyrate	461 (1.1) ^cd^	467 (3.4) ^d^	485 (4.4) ^e^	398 (1.3) ^a^	425 (6.1) ^b^	454 (2.0) ^c^
Ethyl 2-methylbutyrate	10.9 (0.14) ^ab^	11.8 (1.13) ^b^	11.4 (0.63) ^b^	9.7 (0.35) ^a^	11.2 (0.34) ^ab^	20.6 (0.92) ^c^
Ethyl isovalerate	25.9 (1.55) ^bc^	24.3 (0.99) ^bc^	24.8 (0.21) ^bc^	22.5 (0.63) ^b^	19.4 (0.78) ^a^	53.5 (2.12) ^d^
Ethyl valerate	3.60 (0.565) ^ab^	3.90 (0.141) ^ab^	4.40 (0.565) ^bc^	3.30 (0.424) ^a^	5.10 (0.142) ^c^	4.30 (0.422) ^abc^
Ethyl hexanoate	1188 (16.2) ^d^	1254 (21.2) ^e^	1252 (32.5) ^e^	927 (12.7) ^a^	1092 (14.8) ^c^	1034 (31.8) ^b^
Ethyl octanoate	2892 (16.2) ^e^	2716 (26.8) ^d^	2679 (26.2) ^d^	1631 (34.6) ^a^	1972 (23.3) ^b^	2365 (41.7) ^c^
Ethyl 3-hydroxybutyrate	220 (1.4) ^b^	211 (11.3) ^b^	250 (11.4) ^c^	264 (4.9) ^c^	0 (0.0) ^a^	0 (0.0) ^a^
Ethyl 2-hydroxyhexanoate	649 (20.5) ^c^	720 (2.8) ^d^	624 (20.5) ^c^	262 (7.8) ^b^	0 (0.0) ^a^	0 (0.0) ^a^
Ethyl decanoate	95.2 (1.06) ^c^	51.8 (1.13) ^a^	64.6 (1.98) ^b^	120 (4.2) ^d^	310 (2.8) ^e^	402 (5.6) ^f^
Ethyl dodecanoate	105 (5.6) ^c^	96.5 (6.36) ^c^	83.1 (4.38) ^b^	77.1 (2.96) ^b^	17.3 (1.81) ^a^	22.9 (2.99) ^a^
2-phenyl ethanol	9.05 (0.212) ^b^	9.20 (0.141) ^b^	10.5 (0.78) ^c^	5.45 (0.212) ^a^	11.5 (0.78) ^c^	26.0 (0.49) ^d^
Acetate esters (mg/L)						
Isobutyl acetate	50.3 (0.99) ^d^	46.1 (1.20) ^c^	51.7 (0.99) ^d^	38.8 (1.13) ^b^	55.4 (0.84) ^e^	23.6 (0.56) ^a^
Isoamyl acetate	2607 (13.4) ^b^	2712 (35.3) ^c^	2690 (32.5) ^c^	1467 (26.8) ^a^	3343 (33.9) ^d^	1487 (19.1) ^a^
Hexyl acetate	122 (2.1) ^b^	122 (2.0) ^b^	133 (2.2) ^c^	136 (2.2) ^c^	195 (1.4) ^d^	67.0 (1.42) ^a^
Phenylethyl acetate	31.4 (0.84) ^b^	11.6 (0.56) ^a^	41.3 (1.83) ^c^	32.9 (0.14) ^b^	135 (3.5) ^e^	82.6 (1.98) ^d^
Terpenoids (mg/L)						
Alpha-terpineol	43.8 (1.69) ^b^	41.9 (0.14) ^b^	37.9 (1.48) ^a^	42.5 (0.78) ^b^	52.4 (1.99) ^c^	58.1 (1.27) ^d^

Values with different roman letters (a–f) in the same row are significantly different according to Tukey’s post hoc test (*p* < 0.05). W1: *L. thermotolerans* strain Lt1—*S. cerevisiae* strain A6Y10; W2: *L. thermotolerans* strain Lt1—*S. cerevisiae* strain A26Y23; W3: *L. thermotolerans* strain Lt1—*S. cerevisiae* strain Sc1; W4: *S. cerevisiae* strain A6Y10; W5: *S. cerevisiae* strain A26Y23; W6: *S. cerevisiae* strain Sc1.

## Data Availability

The data presented in this study are available in the manuscript.

## Supplementary Materials

The following supporting information can be downloaded at: https://www.mdpi.com/article/10.3390/microorganisms12040786/s1. Figure S1: Sensory description of the wines produced in the present study. For each descriptor, the upper graph presents the overall description, the graph designated ‘A’ by the French panel, and the graph designated ‘B’ by the Greek panel. Different letters above each box column designate statistically significant differences (*p* < 0.05) of the specific attribute between the different wines. The stars located between graphs ‘A’ and ‘B’ designate statistically significant differences between the evaluation panels for the specific attribute of the same wine (*, *p* < 0.05; **, *p* < 0.01; ***, *p* < 0.001; ns, not significant).
