# Supplementary material for: New Insights into the Production of Assyrtiko Wines from the Volcanic Terroir of Santorini Island Using Lachancea thermotolerans
Source: Microorganisms. 2024 Apr 12;12(4):786. doi: 10.3390/microorganisms12040786 (PMC11052215; doi:10.3390/microorganisms12040786)
Supplement: Supplementary file 1 [file microorganisms-12-00786-s001.zip › microorganisms-2944136-SI.pdf]

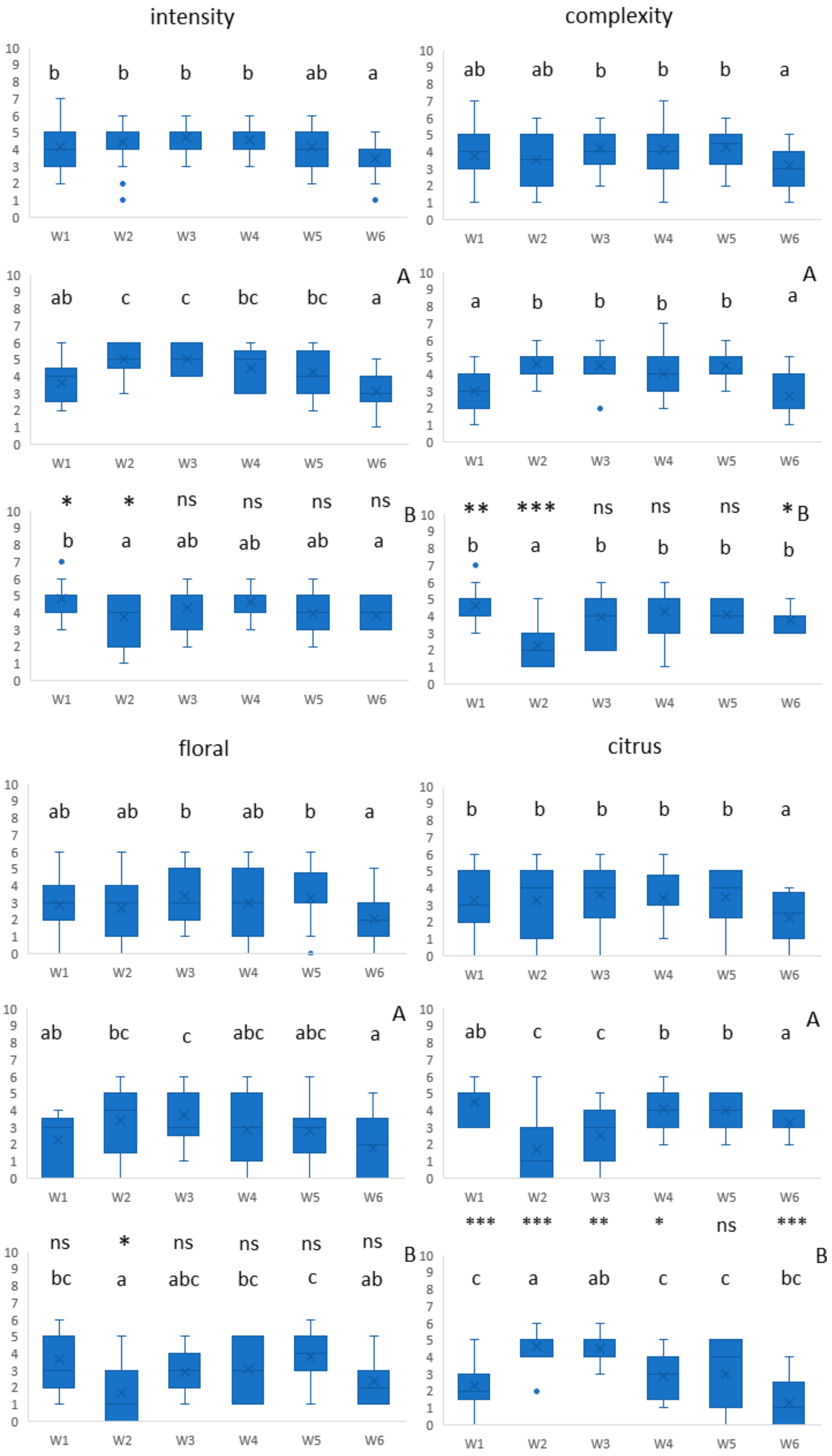

intensity
complexity
floral
citrus
W1 W2 W3 W4 W5 W6
A
B
b b b b ab a
ab ab b b b a
ab c c bc bc a
a b b b b a
* * ns ns ns ns
b a ab ab ab a
** *** ns ns ns *
b a b b b b
ab ab b ab b a
b b b b b a
ab bc c abc abc a
ab c c b b a
ns * ns ns ns ns
bc a abc bc c ab
*** *** ** * ns ***
c a ab c c bc

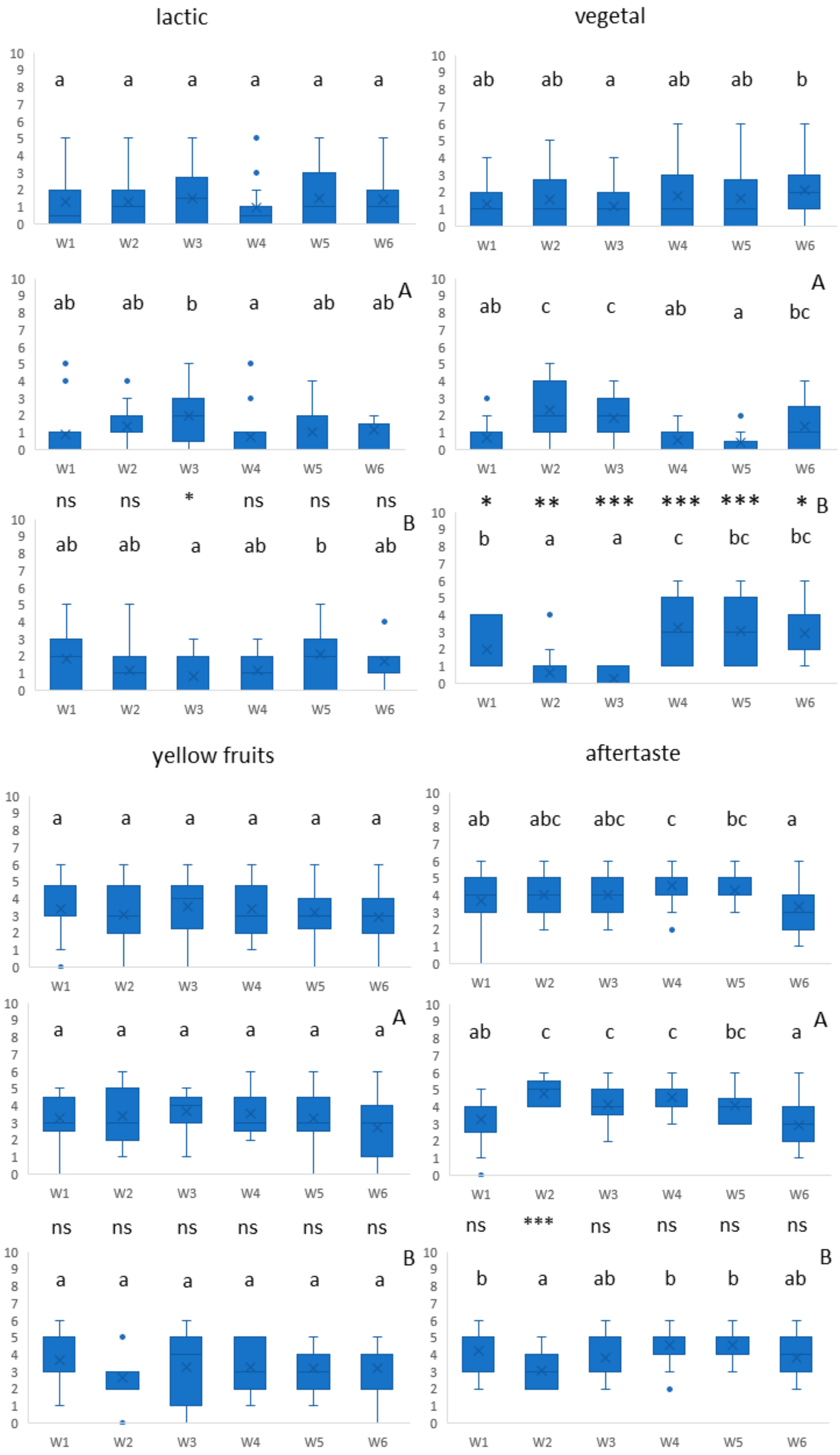
lactic
a a a a a a
W1 W2 W3 W4 W5 W6
ab ab b a ab ab A
W1 W2 W3 W4 W5 W6
ns ns * ns ns ns
ab ab a ab b ab B
W1 W2 W3 W4 W5 W6
vegetal
ab ab a ab ab b
W1 W2 W3 W4 W5 W6
A
ab c c ab a bc
W1 W2 W3 W4 W5 W6
* ** *** *** *** * B
b a a c bc bc
W1 W2 W3 W4 W5 W6
yellow fruits
a a a a a a
W1 W2 W3 W4 W5 W6
a a a a a a A
W1 W2 W3 W4 W5 W6
ns ns ns ns ns ns
a a a a a a B
W1 W2 W3 W4 W5 W6
aftertaste
ab abc abc c bc a
W1 W2 W3 W4 W5 W6
ab c c c bc a A
W1 W2 W3 W4 W5 W6
ns *** ns ns ns ns
b a ab b b ab B
W1 W2 W3 W4 W5 W6

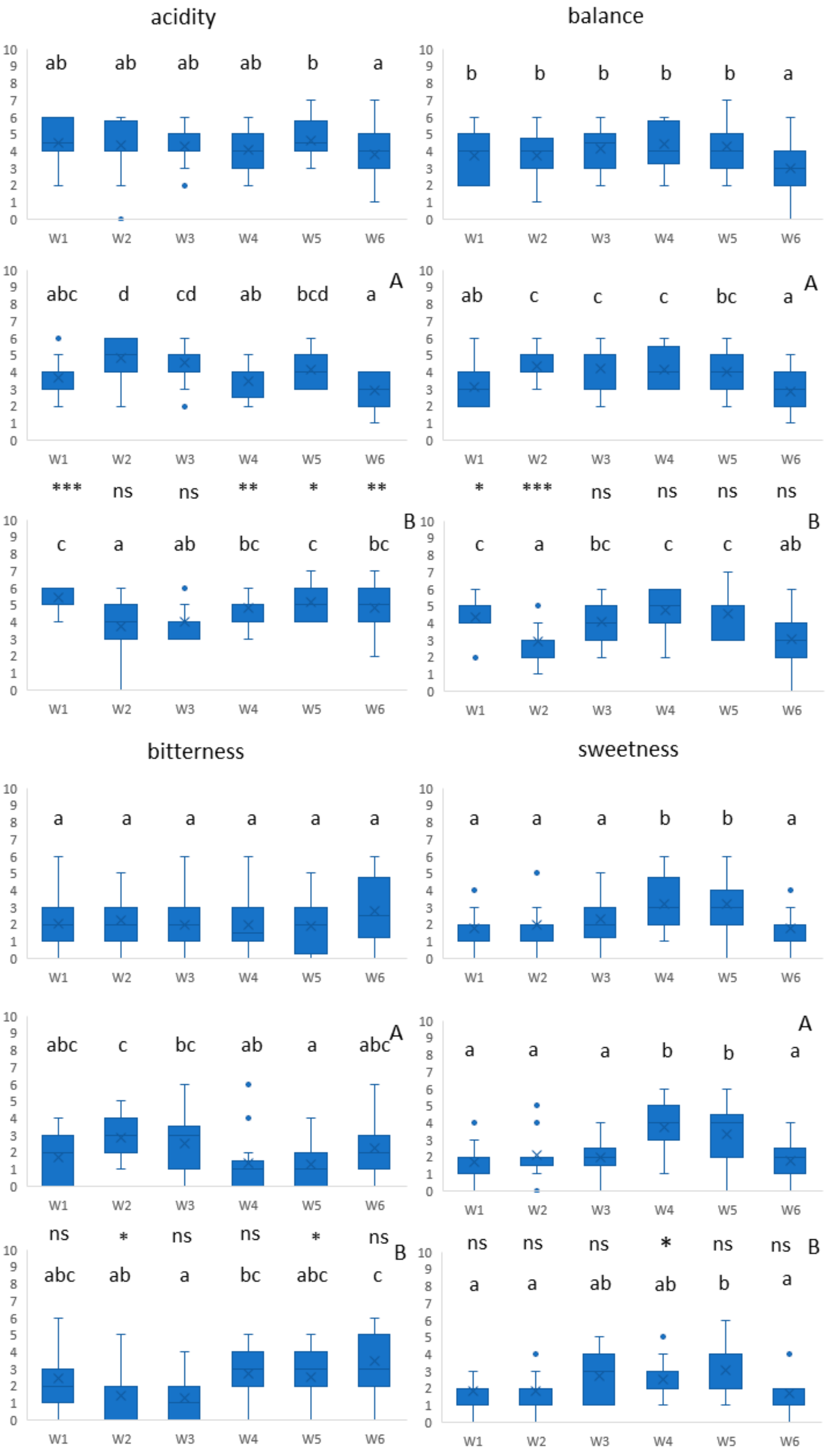

acidity
ab ab ab ab b a
W1 W2 W3 W4 W5 W6
abc d cd ab bcd a
A
*** ns ns ** * **
c a ab bc c bc
B
balance
b b b b b a
ab c c c bc a
A
* *** ns ns ns ns
c a bc c c ab
B
bitterness
a a a a a a
abc c bc ab a abc
A
ns * ns ns * ns
abc ab a bc abc c
B
sweetness
a a a b b a
a a a b b a
A
ns ns ns * ns ns
a a ab ab b a
B

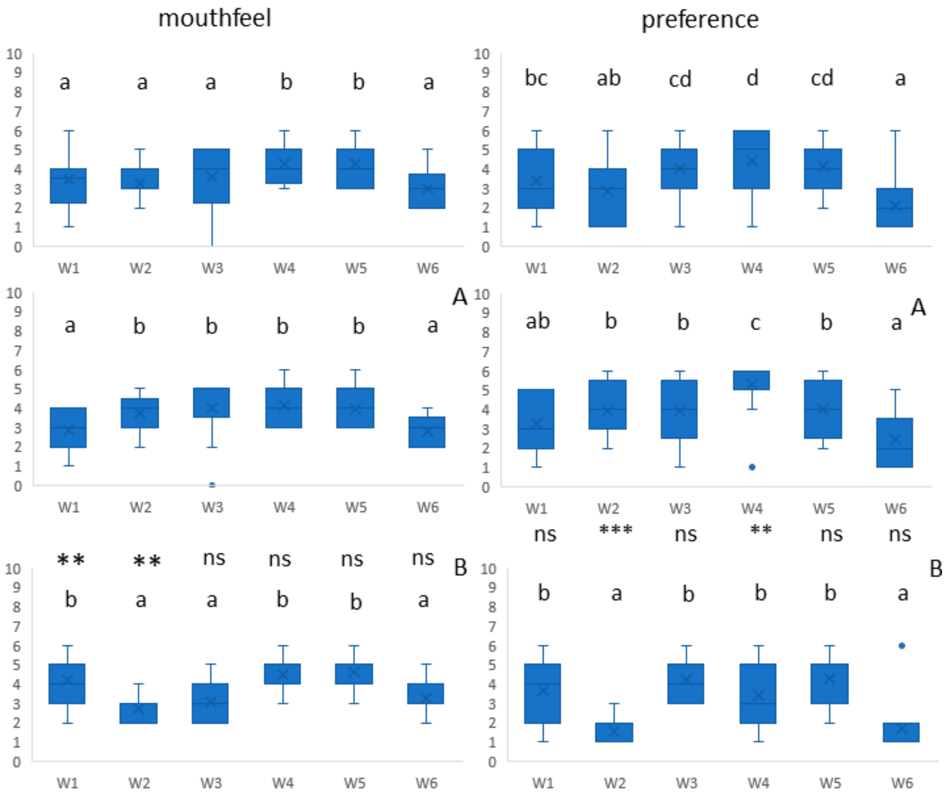

Figure S1. Sensory description of the wines produced in the present study. For each descriptor, the upper graph presents the overall description, the graph designated 'A' by the French panel and the graph designated 'B' by the Greek panel. Different letters above each box column designate statistically significant differences ($p < 0.05$) of the specific attribute between the different wines. The stars located between the graphs 'A' and 'B' designate statistically significant differences between the evaluation panels for the specific attribute of the same wine (*, $p < 0.05$; **, $p < 0.01$; ***, $p < 0.001$; ns, not significant).
